# Phenanthraquinone-Doped Polymethyl Methacrylate Photopolymer for Holographic Recording

**DOI:** 10.3390/molecules27196283

**Published:** 2022-09-23

**Authors:** Jinhong Li, Po Hu, Zeyi Zeng, Junchao Jin, Junhui Wu, Xi Chen, Jie Liu, Qingdong Li, Mingyong Chen, Zuoyu Zhang, Yuanying Zhang, Xiao Lin, Xiaodi Tan

**Affiliations:** 1 College of Photonic and Electronic Engineering, Fujian Normal University, Fuzhou 350117, China; 2 Photonics Research Center, Key Laboratory of Opto-Electronic Science and for Medicine of Ministry of Education, Fujian Provincial Key Laboratory of Photonics Technology, Fujian Provincial Engineering Technology Research Center of Photoelectric Sensing Application, Fujian Normal University, Fuzhou 350117, China

**Keywords:** holographic recording, photopolymer, PQ/PMMA, synthesis, reaction mechanism, PQ/PMMA applications, holographic data storage

## Abstract

Phenanthraquinone-doped polymethyl methacrylate (PQ/PMMA) photopolymers are considered to be the most promising holographic storage media due to their unique properties, such as high stability, a simple preparation process, low price, and volumetric shrinkage. This paper reviews the development process of PQ/PMMA photopolymers from inception to the present, summarizes the process, and looks at the development potential of PQ/PMMA in practical applications.

## 1. Introduction

Holography is a 3D imaging technology that uses the interference principle of light to record the amplitude, the phase, and the polarization information of light waves in the storage media in the form of a hologram and then uses the original reference light to reconstruct the complete recorded information through diffraction. Holography was first proposed by Gabor in 1948 [1]. Due to the limitation of coherent light sources, the early development of holography was slow, but with the emergence and progress of laser technology, holography developed rapidly [2]. As the media of holography, polymer materials have important applications in the fields of three-dimensional holographic display [3,4,5], holographic anti-counterfeiting [6,7,8], holographic data storage [9,10,11], and so on [12,13,14].

The materials used for holographic recording are mainly divided into two categories, organic materials and inorganic materials. Inorganic materials mainly include silver halide, dichromate gelatin, and photorefractive crystals. Silver halide is the earliest material used in holographic recording. It has high sensitivity, a wide sensitivity spectral range, and strong versatility. However, this material has difficult processing and fixing procedures. Dichromate gelatin (DCG) has the characteristics of low scattering rate, high refractive index modulation, and good transparency in the visible. However, DCG sensitivity is relatively low, and the material is sensitive to humidity, which can degrade the hologram if left unprotected. In addition, DCG has a short shelf life that requires the material to be used only a few weeks after production. Photorefractive crystal materials can be rewritten without development, but they never entered large-scale production because of their limited number of applications. Their size is also limited by the growing process, which makes them quite expensive to use. The research on organic materials is mainly focused on polymers, including holographic polymer-dispersed liquid crystals (HPDLCs), azo polymers, photorefractive polymers, photomobile holographic polymers [15,16,17], and photopolymers. We will give a brief overview of these different types of polymer materials in Section 2.

In this paper, the materials that are applied in holographic recording are reviewed. In addition, the characteristics of PQ/PMMA photopolymers, the current research on them, and their applications status are reviewed and discussed. This paper is structured as follows. In Section 2, we introduce some polymer materials for holographic recording. Section 3, Section 4, Section 5, Section 6 and Section 7 introduce the application, material composition and preparation methods, working principles (including thermal- and photopolymerization reaction), optical performance parameters, and test devices of PQ/PMMA photopolymers. In Section 8, we provide an overview of the optimization of PQ/PMMA photopolymers and introduce three research measures to improve the holographic properties. Section 9 provides a brief summary.

## 2. Polymers for Holographic Recording

Polymers as holographic recording media are of great interest due to their inherent advantages such as high refractive index modulation, high photosensitivity, long shelf life, easy to prepare, and low cost. Polymers have been explored by many scholars in the holographic recording field. In this section, we introduce some polymers mainly used for holographic recording, highlight the advantages of photopolymers compared to other polymer materials, and introduce and discuss the PQ/PMMA photopolymer material.

### 2.1. Holographic Polymer-Dispersed Liquid Crystal

Holographic polymer-dispersed liquid crystals (HPDLCs) are a kind of ordered composites with holographic function and are mainly composed of monomers, dispersed liquid crystals, cross-linking monomers, co-initiators, and photoinitiator dyes [18,19]. An HPDLC contains a polymer-rich phase and a liquid-crystal-rich phase in a periodic arrangement and can store the amplitude information of coherent light [20]. Polymerization-induced phase separation under a coherent laser causes the diffusion of the monomer from the coherent dark region to the coherent bright region and the reverse diffusion of liquid crystals, which finally produces holographic gratings periodically arranged by polymer-rich phase and liquid-crystal-rich phase. 

Liquid crystal molecules have unique optical anisotropy, dielectric anisotropy, and excellent ability to respond to an external field. The introduction of liquid crystal molecules into the holographic photopolymer can not only improve the refractive index modulation of the holographic photopolymer but also endow it with optical anisotropy [21]. Hence, HPDLCs have an electro-optical response and high refractive index modulation (10^−2^) and have great application potential in high-end anti-counterfeiting, augmented reality, holographic display, and other high-tech fields [22,23,24]. However, HPDLCs suffer from insufficient stability and a mismatch between the photopolymerization reaction rate and the liquid crystal diffusion rate, which limits the performance and versatility of HPDLCs.

### 2.2. Azo Polymer

Azo polymers are holographic polymer materials composed of photoisomeric molecules and have both cis and trans isomer structures [25,26,27], which can be converted into each other when irradiated by light of specific wavelengths. Azo materials are easy to combine with modified components to form azo supramolecular polymers, azo compound liquid crystals, and so on [28]. When azo-benzene is introduced into the liquid crystal system, the photoinduced change in the azo-benzene molecular shape changes the arrangement of the surrounding liquid crystal molecules, which means that the change in the molecular arrangement will be amplified due to cooperative motion, which can realize image storage in the early stage [29].

Azo-based polymers have polarization holographic properties since their photochromism is caused by the cis-trans isomerization of molecules under the beam. When different polarization state beams are applied to the material, the molecules will reorient, making the material anisotropic and allowing the writing and erasing of information. The change process shows the property of birefringence, and a change in light-induced birefringence can record the light field polarization state. Although azo polymers are extremely sensitive to polarization, the polarization is mostly stored in the form of molecular reorientation, which is disrupted by thermal agitation, making azo polymers susceptible to the surrounding environment. Thermal molecular redistribution erases the recorded hologram over time, which make azo polymers not suitable for long-term applications, such as data storage.

### 2.3. Photorefractive Polymer

Photorefractive polymers originate from the photorefractive property of materials [30]. In photorefraction, when photoelectric materials are irradiated by light, due to electro-optic nonlinearity, their refractive indices change with the spatial space charge field distribution of light intensity. This optical phenomenon can occur in photoconductive effect substances, which are called photorefractive materials. Photorefractive polymers, composed of chromophores, photosensitizers, polymers, and plasticizers, exhibit high-resolution and volume hologram capabilities. The dynamic and reversible nature of the photorefractive polymer allow new light exposure to erase an existing hologram and record another hologram without reducing efficiency or resolution. This material opens the door of holography and makes it rewritable for applications ranging from dynamic imaging of scattering media to renewable three-dimensional (3D) displays [31].

Photorefractive polymers have a wide wavelength response range (from visible to infrared light), have high refractive index modulation, and can be rewritten, but they are less stable and are not suitable for permanent information storage.

### 2.4. Photopolymer

Photopolymers play an important role in holographic recording due to their unique advantages, which are simpler and cheaper to prepare than HPDLCs, azo polymers, and photorefractive polymers. Photopolymers also have a wide spectral response range, high stability, and long storage life, and they can be widely used in holographic data storage, sensing, light shaping, and security fields. 

A photopolymer is usually composed of one or more monomers, initiators, photosensitizers (dyes), and a polymer matrix [32]. The use of a photopolymer as a holographic storage material is based on its photopolymerization effect [33], which means that under illumination, the photosensitizer can absorb the photons of the corresponding sensitive wavelength and be excited to a higher energy level, and an excited photosensitizer would produce free radicals, which trigger the polymerization of monomers to produce photoproducts [34]. In addition, the polymerization process is related to light intensity. During holographic recording, as a result of the interference between the coherent signal light and the reference light, the light intensity in the exposure field produces non-uniform distribution, which leads to the photopolymerization of monomers and the generation of photoproducts by the photosensitizer in the bright region, generating a refractive index difference. Since the refractive index of the polymerized region (brightly striped region) and the non-converged region (dark-striped region) is modulated, the bit phase type raster modulated by the refractive index modulation can be recorded [35]. According to the substrate-film-forming components, photopolymers can be roughly divided into categories such as polyvinyl alcohol and acrylamide (PVA/AA) photopolymers [36,37,38,39], epoxy resin photopolymers [40,41,42], and phenanthraquinone-doped polymethyl methacrylate (PQ/PMMA) photopolymers. PQ/PMMA photopolymers have some unique advantages. For example, they can be cast into millimeter-thick films, which allows for angular and spectral multiplexing; the large thickness and low index modulation help to achieve high spectral and angular selectivity for multiplexing; they are low cost and have a simple preparation process; and photoinduced shrinkage is negligible. In addition, they can be widely used in holographic data storage, polarized holographic and other fields, making them an attractive study material for holographic recording. However, it is worth mentioning that PQ/PMMA has low refractive index modulation and photosensitivity. So, improving these properties is crucial to optimizing its application in holographic recording. In this study, we look the development of PQ/PMMA photopolymers in detail.

## 3. Applications of PQ/PMMA Photopolymers

As bulk holographic recording materials, PQ/PMMA photopolymers can be widely used in the field of holographic recording, especially in holographic data storage. Multiplexing technologies improve the storage capacity of storage systems, endowing PQ/PMMA with great application potential in the big data era. The principle of holographic data storage, several common multiplexing techniques, and polarization holography are described below.

### 3.1. Principle of Holographic Data Storage

#### 3.1.1. Recording

**Figure 1:**
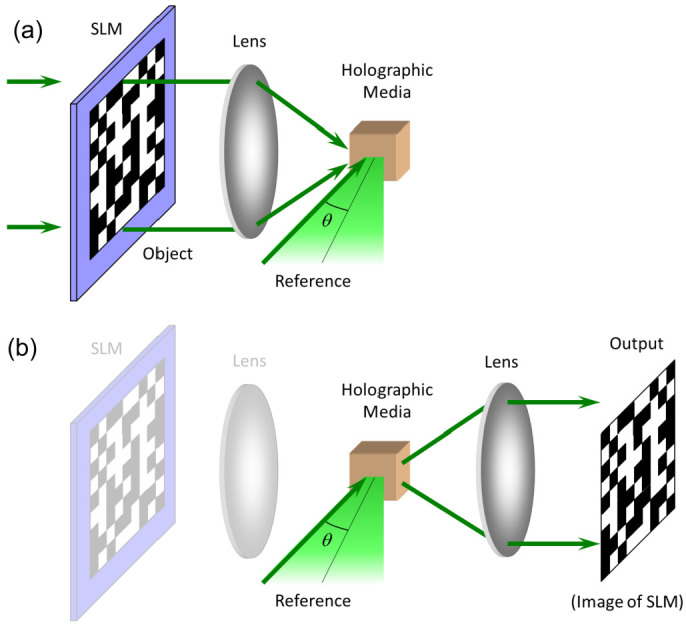
The holographic data storage (**a**) recording and (**b**) reading schematics.

In the holographic data storage recording stage, a coherent laser beam is divided into two parts: A signal beam (carrying data) and a reference beam. The data are encoded onto the object beam by a spatial light modulator (SLM). The data to be stored are binary coded to obtain a two-dimensional data page; then, they are encoded onto the object beam by an SLM and converted into a shaded pixel array. When the object beam and the reference beam interfere in the holographic media, the hologram is recorded in the interference region. By changing the angle of the reference beam or the position of the media, hundreds of unique holograms are recorded in the same volume of material. The recording process is as shown in Figure 1a.

#### 3.1.2. Reading

The reproduced reference light irradiates onto the data storage recording media. Then, the previously recorded pattern can be diffracted. A photoelectric detector converts the diffraction pattern into an electrical signal, which is output by decoding, producing the original stored data. By varying the reference beam, such as angle or wavelength, many different data pages can be recorded in the same volume of material and read out by the recording reference beam. Multiplexing technology enables holographic storage and has great potential for storing high-capacity data before the photosensitizer is exhausted. Compared with the SSD (Solid state disk, 1 TB, 500 MB/s) and BD (Blu-ray disc, 100 GB, 128 MB/s) technologies, the holographic data storage and data transfer rate can reach values of 1.6 TB and 1 GB/s, respectively; the storage recording density can reach a level of TB/in^2^ (1 in = 2.54 cm); and the data reading conversion rate can be as high as 10 GB/s [43]. The reading process is presented in Figure 1b.

### 3.2. Multiplexing Technology

**Figure 2:**
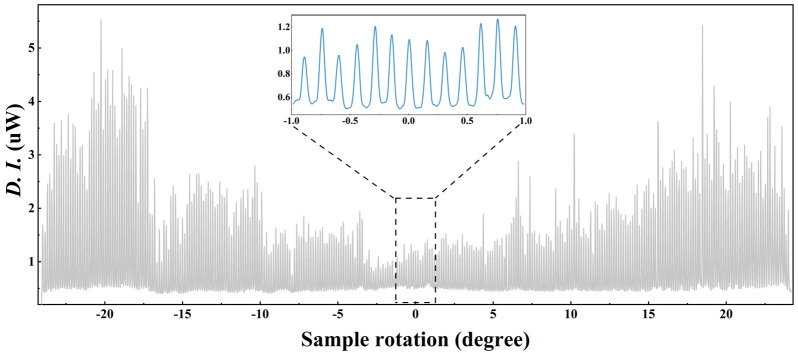
The multiplexing of 321 weak holograms within the C_60_-PQ/PMMA photopolymer [46].

The high diffraction efficiency of PQ/PMMA photopolymers allows them to be combined with multiplexing technology to increase their storage density in holographic data storage. At present, the main holographic data storage multiplexing technologies are divided into the following categories:

- Angle multiplexing [44,45] uses signal light and reference light to record at the same wavelength and different reference light angles with the material in the same position and generates multiple holograms with multiple signal lights. For a 1.5 cm thick doped modified PQ/PMMA photopolymer, 532 nm green light was used as the recording and reference light, with an angular selectivity of roughly 0.15°, achieving a 321-frame hologram recording in a −24 to 24° range [46], as shown in Figure 2,
- Shift multiplexing [47,48] helps to separate and multiplex different holograms in space; that is, after recording a hologram somewhere, it can move a certain distance and then record the next hologram. It takes advantage of the sharp decrease in the diffraction efficiency after Bragg condition mismatch, and the number of multiplexes is limited by separating the hologram in the position space. Shift multiplexing technology is mostly used in photopolymer materials, which can work well with optical disk holographic data storage systems, and it is a highly compatible multiplexing method,
- Wavelength multiplexing [49] uses planar waves with different wavelengths as recorded light and reference light, thereby recording and generating multiple holograms in a material. Photopolymer materials have different levels of sensitivity to different wavelengths, and the response is not a simple linear relationship. Wavelength multiplexing is generally suitable for inorganic photorefractive crystal materials,
- Phase-coded multiplexing [50,51] involves the use of reference light of the multiplexed hologram to perform phase coding so as to avoid crosstalk to the maximum extent. In particular, orthogonal-phase multiplexing of reference light can effectively suppress inter-page crosstalk,
- In polarization multiplexing [52], polarized light in different polarization states is used as a recording light and a reference light to generate multiple holograms, which requires the recording material to have polarization sensitivity.

These multiplexing technologies can be combined with each other and widely used in holographic storage systems to further increase storage density. The existing mature research on holographic storage systems is mainly on off-axis holographic storage systems and collinear holographic storage systems. The biggest difference between these two systems is whether the optical path of the signal light and the reference light is collinear [53]. This paper mainly describes the application of PQ/PMMA in collinear holographic storage systems.

### 3.3. Collinear Holographic Storage System

**Figure 3:**
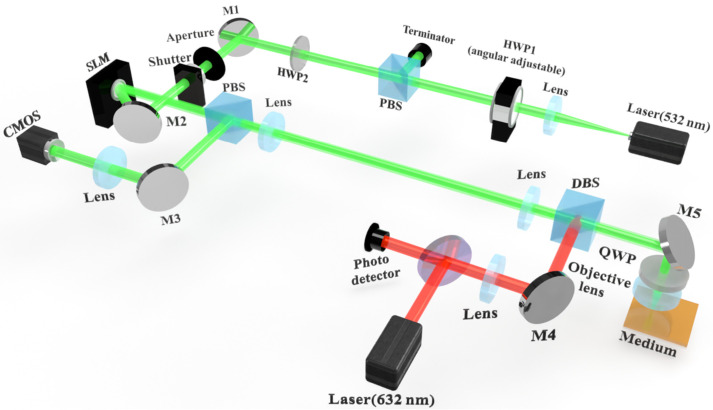
Schematic of the collinear holographic storage system. HWP: half-wave plate; QWP: quarter-wave plate; PBS: polarization beam splitter; PD: photodetector; M: mirror; SLM: spatial light modulator; CMOS: complementary metal oxide semiconductor.

The high stability and low volumetric shrinkage of PQ/PMMA photopolymer allow it to be widely used in collinear holographic storage systems. The collinear holographic storage system [54] was proposed by Japan’s Optware Company, Atsugi, Japan, and Figure 3 displays the optical path diagram.

This system uses the same spatial light modulator (SLM) to simultaneously modulate both the signal light and the reference light on the same axis. In detail, the signal light containing the modulation information is set in the center, and the reference light is set at the outer circle of the signal light. In the recording process, the two beams are simultaneously focused on the holographic recording material and interfere. Then, the hologram is recorded in the material. Note that the images are loaded with the same polarized signal and reference lights. In the reading process, the original reference beam pattern is loaded onto the SLM, and after irradiating the recording material, the signal image can be reproduced. The quarter-wave plate placed in front of the microscope objective can convert the incident p-pol light into circularly polarized light because the diffraction efficiency obtained with circular polarization is better with this material. On return from the reflection layer of the holographic disc, the circularly polarized light passes through the quarter-wave wave plate again and the diffracted light is converted from circularly polarized light to s-pol light. When the s-pol light passes through the polarized beam splitter, it is fully reflected and received by the CMOS without influencing the incident beam.

The red laser and related parts are the servo controllers used between the microscope objective and the holographic material. Since the holographic recording material used is not sensitive to red light, the red laser is used as the light source of the servo system, which can monitor the dynamics of the recording material in real time. The reflected red light can be captured by the photodetector to determine whether the material is defocused, tilted, translated, etc., and the objective lens can be controlled to compensate according to the feedback.

### 3.4. Polarization Holography

**Figure 4:**
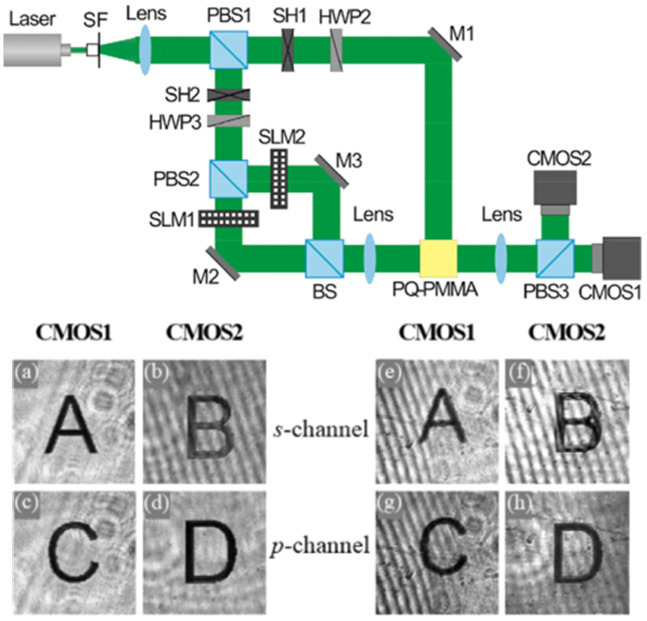
Optical setup of four-channel polarization holographic recording and the experimental reconstructed images [63]. Images reconstructed in four-channel holographic image recording. (**a**–**d**) are original transmitted images before holographic recording. Reconstructed image by the (**e**,**f**) s-polarized reading wave and by the (**g**,**h**) p-polarized reading wave.

The polarization property of PQ/PMMA photopolymer also distinguishes it from other photopolymers. In traditional holography, because the intensity distribution of two interference waves, which include amplitude and phase, is recorded, only the same components of the polarization state of the two interference waves are considered. The actual polarization states of two interference waves are ignored. In polarization holography, not only the amplitude and phase of two waves but also the polarization states of two waves are recorded [55,56,57,58]. For this reason, polarization holography is expected to have more abundant characteristics of reconstruction and a wide range of applications [59,60]. A polarization-sensitive recording material is the key to research on polarization holography [61]. Currently, PQ/PMMA photopolymers can record polarization holography [62], and the four-channel polarization multiplexing has been implemented by using these polymers [63]. The optical path diagram and experimental results are shown in Figure 4. Dual-channel multiplexing of left- and right-handed circular polarization light is also realized [64,65]. Polarization holography adds the polarization information of light as a modulation dimension to the holographic storage, which can increase the information storage density. As per research in recent years [66], polarization holography is a good candidate to replace the existing micro–nano technology [67], for the manufacture of light field control elements [68,69], and for the manufacture of metamaterials [70]. The polarization characteristics of the PQ/PMMA photopolymer originate from the optical anisotropy within the material, and we will describe its measurement optical path in detail in Section 7.

The holographic properties of PQ/PMMA photopolymers considerably enhance their data storage capacity and enable their application in bulk holographic data storage. Thus, PQ/PMMA shows great potential for holographic data storage.

## 4. PQ/PMMA Materials and Preparation

**Figure 5:**
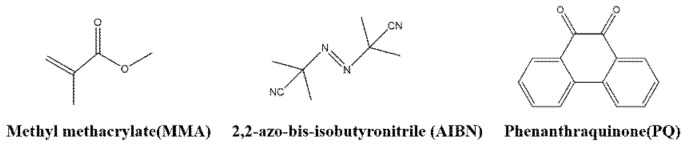
Schematic diagram of the chemical composition of PQ/PMMA photopolymers.

Phenanthraquinone-doped polymethyl methacrylate (PQ/PMMA) photopolymer materials are usually prepared by the thermopolymerization method [71]. PQ/PMMA is composed of monomer methyl methacrylate (MMA), heat initiator 2,2-azo-bis-isobutyronitrile (AIBN), and photosensitizer phenanthraquinone (PQ). The chemical structure of these components is shown in Figure 5.

**Figure 6:**
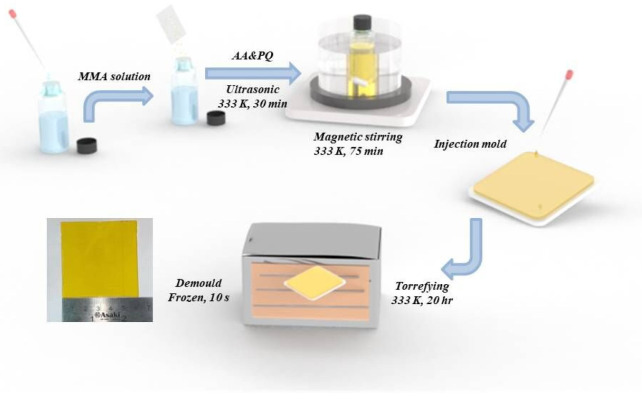
Schematic synthesis strategy of PQ/PMMA photopolymer materials.

PQ/PMMA photopolymers are prepared by thermal polymerization. Figure 6 displays the two-stage polymerization process of PQ/PMMA:

(1) The weighted MMA is added to a transparent glass bottle,
(2) The PQ photosensitizer (1.0 wt %) and the AIBN thermo-initiator (1.0 wt %) are added. Their proportion in the mixture is maintained at MMA:AIBN:PQ = 100:1:1,
(3) All the components are ultrasonically shaken in a water bath at 333 K for 20 min to form a homogeneous multi-component solution,
(4) During stirring pre-polymerization, the glass bottle is placed on a magnetic stirrer and kept at a constant temperature (333 K) for 75 min to make each solution homogeneously viscid,
(5) The viscous solutions are poured into glass molds with spacers of a specific thickness, and the glass molds are placed horizontally in an oven at 333 K for 20 h until complete solidification. Finally, a yellow transparent solid PQ/PMMA material is obtained. The thickness of the PQ/PMMA material can be regulated by controlling the thickness of the spacers.

## 5. PQ/PMMA Operational Principle

### 5.1. Thermal Polymerization

During the preparation of the material, from the stirring of each monomer component on a constant temperature (60°) magnetic stirrer to the formation of a yellow transparent solid PQ/PMMA material, a thermal polymerization reaction occurs. During this reaction, the MMA monomer molecules produce thermal polymerization under the initiation of the thermal initiator azo-bis-isobutyronitrile (AIBN), and most MMA molecules polymerize into chains to form a PMMA polymer substrate. Specifically, the reaction can be divided into the following steps:

#### 5.1.1. Chain Initiation

**Figure 7:**
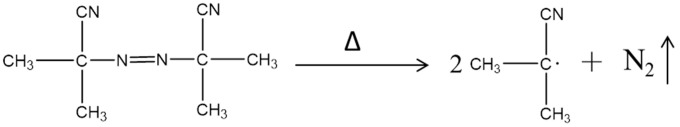
The decomposition of AIBN.

**Figure 8:**
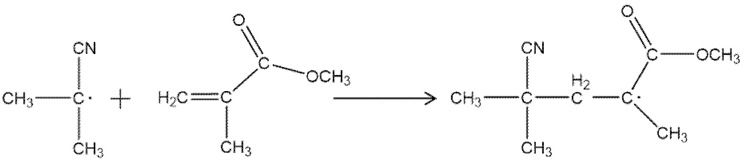
Free radical formation.

The N = N double bond in the thermal initiator AIBN is heated and fractured, and the covalent bonds are evenly cracked to produce two primary radicals and N2, as shown in Figure 7. Then, the two free radicals react with the MMA monomer molecule to form monomer free radicals, as shown in Figure 8.

#### 5.1.2. Chain Growth

**Figure 9:**
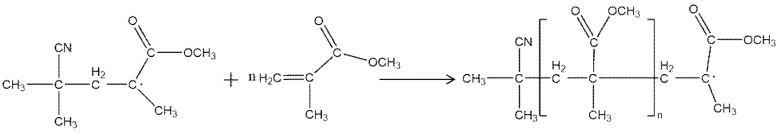
Monomeric radical chain additions form long-chain radicals.

The chain-initiation-stage-produced monomer free radicals have extremely high activity and can occur in the chain addition reaction with other MMA monomer molecules, as shown in Figure 9. Monomer radicals bind to countless monomer molecules to form long-chain radicals, which is a strongly exothermic reaction with a fast reaction rate.

#### 5.1.3. Chain Termination

**Figure 10:**
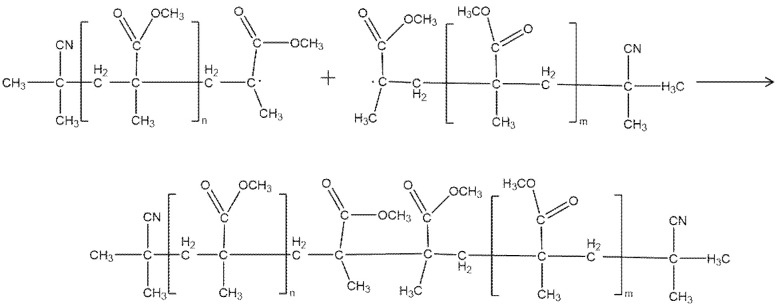
Coupling termination products.

**Figure 11:**
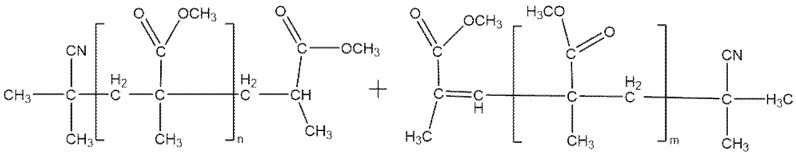
Disproportionation termination products.

Different long-chain free radicals are highly active, cannot exist alone, and will interact with each other and terminate. There are two forms of chain termination: coupling termination and disproportionation termination. The products of these two different terminations are shown in Figure 10 and Figure 11, respectively.

### 5.2. Photoreaction Process

During holographic recording, the coherent object and the reference light interfere with each other to generate periodic light field distribution between light and dark. At this time, photopolymerization occurs in the bright region of the photopolymer, and little or no reaction occurs in the dark region, which results in a gradient difference between the refractive index of the bright and dark regions of the material, thus forming holographic gratings [35,72].

**Figure 12:**
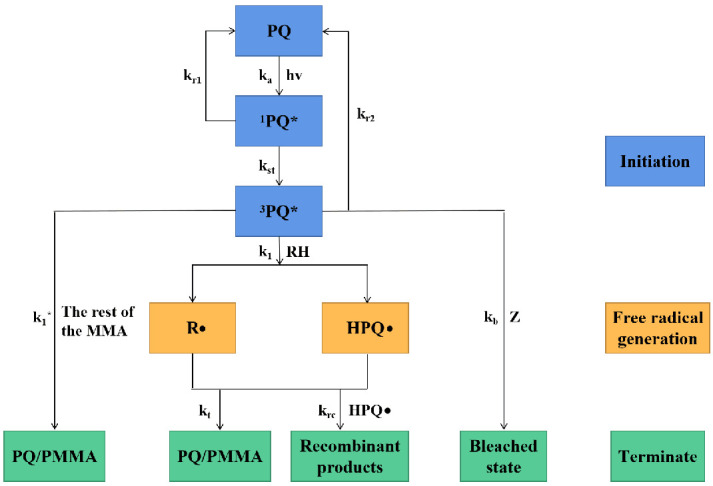
Photopolymerization reaction mechanism process, * represent the excited state of PQ.

Under light illumination, the photosensitizer PQ absorbs photon energy, changing from the ground state to a single excitation state 1PQ* and gradually converting to a more stable triple excitation state 3PQ*. The 3PQ* molecule carbonyl (C = O) functional group is broken, reacts with the C = C double bond of the nearby PMMA polymer chain RH, takes a hydrogen atom from the methyl group, and finally forms the semiquinone radical HPQ• and the polymeric radical R•. The two generated free radicals are active, and the unsaturated vinyl group in R• reacts with HPQ• one-to-one to form the photoproduct PQ-PMMA. In addition, the triple-excited-state 3PQ* reacts with the remaining MMA molecules to form another photoproduct, PQ-MMA. Figure 12 presents a diagram of the whole mechanism of photopolymerization reaction.

During the photopolymerization reaction [73,74], the PQ photosensitizers, the MMA monomers, and the PMMA long chains in the bright region generate PQ-PMMA and PQ-MMA photoproducts. At this time, the resulting photoproducts are mainly in the bright region, while in the dark region, because there is almost no chemical reaction, there are still high concentrations of unreacted PQ molecules and MMA and PMMA polymers. Because of the modulation and the concentration gradient of the chemical composition in the bright and dark regions, PQ molecules and MMA monomers diffuse from the dark region to the bright region, and it is difficult for the photoproduct macromolecules to diffuse to the dark region in a short time, resulting in the average concentration gradient difference of different chemical components in the light and dark regions, which produces a change in refractive index modulation by the material [75,76]. Therefore, in PQ/PMMA photopolymers, the refractive index modulation of the grating consists of two parts: one contributed by the photochemical reaction during the exposure and the other contributed by the diffusion of the components after the end of the exposure. Temperature affects the diffraction efficiency, the diffusion of components, and the polarization properties of the material, so the choice of temperature in the photopolymerization reaction is crucial. In addition, chain growth and chain length during the photopolymerization of polymer materials cannot be neglected, and the presence of chain length is considered a non-local effect [77,78,79]. The application of the non-local model can more accurately describe the real chain growth process of the material during the exposure.

## 6. Optical Performance Characterization Parameters of PQ/PMMA

### 6.1. Ultraviolet Absorption Spectrum

**Figure 13:**
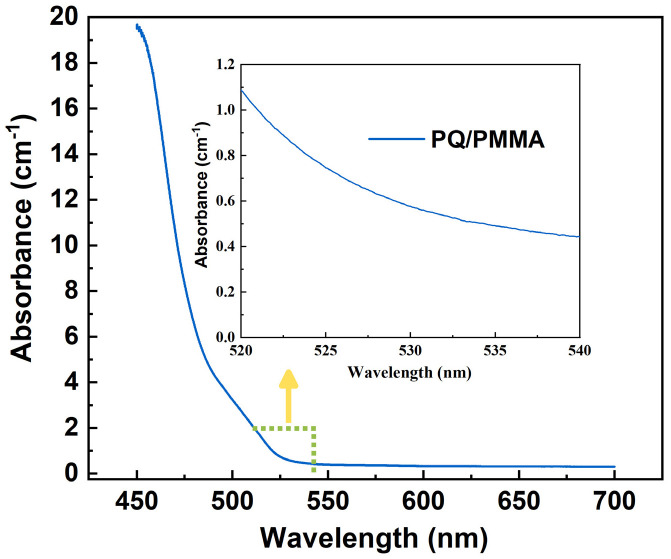
UV-vis absorption spectrum of 1.5 mm PQ/PMMA.

Ultraviolet absorption spectroscopy can effectively test the absorption capacity of materials in specific light bands. In the holographic recording process, the material needs to absorb a certain amount of light for photopolymerization reaction, but the absorption value should not be too high. The ultraviolet absorption spectroscopy of the 1.5 mm thick PQ/PMMA photopolymer was measured using a UV-vis spectrophotometer, as shown in Figure 13. 

The UV spectrophotometer test absorption value formula can be expressed as:

(1) $$A=-\ln\frac{I_{t}}{I_{f}}$$

where *I_f_* and *I_t_* are the intensities of the incident and transmitted light, respectively. Note that the effect of the reflection of materials on recorded light was ignored here.

### 6.2. Diffraction Efficiency

The diffraction efficiency curve of a grating can reflect the speed and amplitude of grating formation. Usually, there are two ways to express diffraction efficiency:

(1) Define diffraction efficiency *η*1 (external diffraction efficiency) as the ratio of the effective diffraction flux of first-level diffraction to the effective incident luminous flux of grating reproduction diffraction.
(2) Ignore the effect of reflection and absorption of materials on recorded light and define the diffraction efficiency *η*2 (internal diffraction efficiency) as the ratio of reproducing diffracted light intensity to incident light intensity. The calculation formulas for *η*1 and *η*2 are as follows:

(2) $$\eta 1=\frac{I_{+1}}{I_{\mathrm{in}}}$$

(3) $$\eta 2=\frac{I_{+1}}{I_{0}{+I}_{+1}}$$

where *I*_0_, *I*_+1_, and *I*_in_ express the intensities of the transmitting beams, the first-order grating diffraction beams, and incidence light intensity of incident beams, respectively.

### 6.3. Photosensitivity

Photosensitivity can reflect the ability of a material to form a raster: that is, the ability to read and write holographic records. It defines *S* as the slope of the square root of the diffraction efficiency of a material at a given moment multiplied by the incident light intensity and material thickness [80,81]:

(4) $$S=\frac{1}{\mathrm{Id}}\left(\;\;\frac{\partial \sqrt{\eta }}{\partial t}\;\right)$$

where *I* is the intensity of the recording wave, *d* is the thickness of the material, and *η* is the diffraction efficiency.

### 6.4. Refractive Index Modulation

Refractive index modulation can reflect the grating strength and the legibility of the reconstructed hologram. According to Kogelnik’s coupled wave theory [82], we can obtain the relationship between the grating diffraction efficiency *η* and the refractive index modulation Δ*n*(*t*) of the material, which can be divided into two categories:

For a transmission hologram:

(5) $$\Delta n\left(t\right)=\frac{\lambda \cos\theta_{0}}{\pi d}\arcsin\sqrt{\eta }$$

For a reflection hologram:

(6) $$\Delta n\left(t\right)=\frac{\lambda \cos\theta_{0}}{\pi d}\mathrm{arctanh}\sqrt{\eta }$$

where *λ* is the recording information wavelength (532 nm), *θ*_0_ is the Bragg angle corresponding to the recorded light, *d* is the thickness of materials, and *η* is the diffraction efficiency at the Bragg angle position.

### 6.5. Dynamic Range

The storage capacity of a material is evaluated by keeping the material in the same spatial position and reading the maximum diffraction efficiency of all recorded gratings, corresponding to its maximum refractive index change amplitude, which is called the dynamic range *M*^#^. For different gratings with different diffraction efficiencies, the dynamic range *M*^#^ can be defined as the sum of the square root of the diffraction efficiency of all recorded gratings [72]. The formula is expressed as:

(7) $$M^{\#}=\overset{N}{\underset{i=1}{\sum }}\sqrt{\eta_{i}}$$

where *N* is the maximum number of gratings that can be recorded by the material. 

### 6.6. Volumetric Shrinkage

The volumetric shrinkage of photopolymers is a crucial factor in holography data storage as it causes significant grating distortion and Bragg shift and consequently the failure of data reading [83]. The value of volume shrinkage *σ* is calculated as:

(8) $$\sigma =1-\frac{\tan\theta_{\mathrm{theo}}}{\tan\theta_{\exp}}$$

where *θ*_theo_ and *θ*_exp_ represent the main lobe Bragg angle positions in theory and experiment after exposure, respectively. 

## 7. Holographic Test Device

To more scientifically and accurately describe the holographic recording characteristics of PQ/PMMA photopolymers, this section provides details of the experimental test device for PQ/PMMA holographic performance, including the test device for diffraction efficiency and the test device for photoinduced birefringence.

### 7.1. Diffraction Efficiency

**Figure 14:**
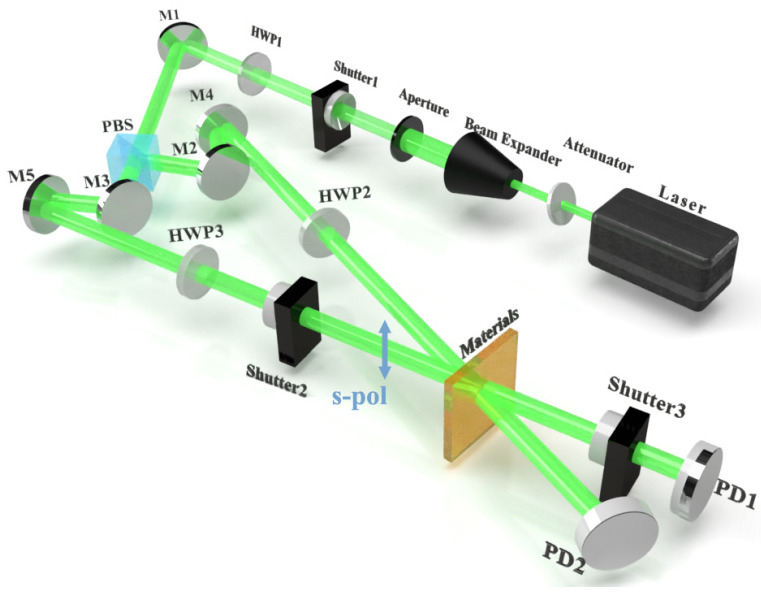
Experimental setup for diffraction efficiency measurement. HWP: half-wave plate; PBS: polarization beam splitter; PD: photodetector; M: mirror.

The experimental tests of holographic diffraction characteristics use a symmetrical two-optical path interferometry device, as shown in Figure 14.

The intensity of the beam emitted by the 532 nm green laser is first attenuated by the attenuation sheet, the spot size is adjusted by beam expansion and the diaphragm, and the beam passes through a half-waver HWP1. Then, it is reflected by the planar mirror M1 and becomes divided into a vertically polarized reference beam s-pol and horizontally polarized object beam p-pol, where the ratio of the intensities of object and reference beams incident on the material can be adjusted using HWP1. The polarization state of the reference beam is regulated by controlling HWP2 only in polarization holography. The polarization state of the reference beam s-pol changes to p-pol after it passes through HWP2. In conventional intensity holography, the reference beam is set to s-pol. The object beam p-pol passes through HWP3 and adjusts its polarization state consistent with the reference beam. During recording, shutter 1 is always open, shutter 2 is open for 6 s, and the object beam and the reference beam record the grating on the material. During reproduction, shutter 2 is closed and shutter 3 is open for 0.4 s. Reproduction grating is performed with the original reference beam, and the intensities of transmitted and diffracted light are measured by the PD. These two processes are repeated until the grating reaches saturation.

### 7.2. Photoinduced Birefringence

**Figure 15:**
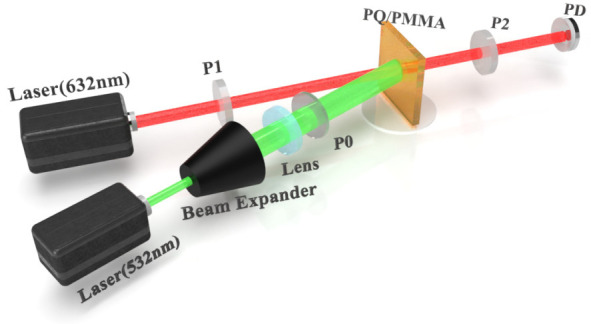
Experimental setup of photoinduced birefringence measurement. P0, P1, and P2 stand for the horizontal polarizer, the negative 45° polarizer, and the positive 45° polarizer, respectively.

A PQ/PMMA photopolymer has polarization property [66,84]. Photoinduced birefringence is a macroscopic embodiment of the microscopic molecular anisotropy of the medium and is an important photophysical parameter of anisotropic materials. Figure 15 displays the experimental setup of the photoinduced birefringence measure.

Before exposure, the sample as a whole is isotropic and does not respond to the polarization characteristics of the probe light. In addition, the probe light cannot pass through the two orthogonal polarizers of the measurement system. When the sample is exposed to linearly polarized pump light, a photobirefringence effect occurs. The intensity of the transmitted light of the system is related to the photobirefringence of the sample and increases with an increase in photobirefringence. The transmittance signal of the monitoring system can be used to measure the photobirefringence of the material. 

In the experimental optical path system, the angle between the two beams should be as small as possible. To trigger the birefringence phenomenon, the red light at 632.8 nm is used as the detection light, since the material does not absorb light of this wavelength, and the green light at 532 nm is used as the pump light, since the material absorbs light of this wavelength. The laser at 632.8 nm is incident on the material via a horizontal polarizer after passing through the beam expansion system. The 632.8 nm laser is incident on the material through a negative 45° P1 polarizer and then through a positive 45° P2 polarizer, which is received by the photodetector. To better measure the change in the refractive index of the material, the spot of the pump light has to completely cover the spot of the probe light [85]. The value of the birefringence is calculated as:

(9) $$\Delta n\left(t\right)=\frac{\lambda }{\pi d}\arcsin\sqrt{\frac{I_{T}}{I_{0}\sin^{2}2\theta }}$$

where *I_T_* is the intensity of the detected light incident on the photodetector, *θ* is the angle between the polarization direction of the pump light and the probe light, *I*_0_ is the incident intensity of the probe light, *d* is the thickness of the material, and *λ* is the wavelength of the recording light. To simplify the calculation, the *θ* is set to be 45° here.

## 8. Origins of PQ/PMMA and Performance Improvement Measures

PQ/PMMA photopolymers of thicknesses between 100 and 200 μm were first proposed by Veniaminov in 1996 [86]. The diffusion coefficients of PQ molecules in different media (viscous liquid, rubber state, and glass state) were measured by holography at temperatures of 20~270 °C, and the measured range was 10^−21^~10^−11^ M^2^/s. This provides experimental guidance for preparing PQ/PMMA photopolymers in the later stage and also lays the cornerstone for future research on PQ/PMMA materials. 

The holographic storage characteristics of photopolymer PQ/PMMA materials was first tested by Steckman et al. in 1998 [71]. They found that the material dissolves the PQ molecule and the thermal initiator in the liquid methyl methacrylate. They obtained a sample with a thickness in millimeters by high-temperature and high-pressure thermal polymerization and explained in brief the physical mechanism involved in the recording process of the material. They believed that the PQ molecule reacts with the substrate PMMA after being excited by light to generate PQ/PMMA light products. However, the sensitivity and refractive index modulation system of the PQ/PMMA material they obtained was still poor. With the advent of PQ/PMMA photopolymers for holographic storage, researchers have worked to further improve the holographic properties of materials through various means. 

Lin et al. first proposed the kinetics of the PQ/PMMA photopolymer recording grating formation process in 1999 [87]. On the basis of dynamic measurement, they proposed two exposure schemes that realize multiple storage of equal-intensity rasters: the predetermined exposure method and the incremental exposure method. Then, they realized the storage of multiple planar wave holograms with uniform diffraction efficiency, keeping the PQ/PMMA in the same position.

Subsequently, Lin studied the effect of temperature on the formation of PQ/PMMA photopolymer gratings [88], proposed that the main limitation of recording hologram refractive index modulation is caused by scattering effects, and demonstrated that dark enhancement under exposure can improve the diffraction efficiency of gratings.

To increase the effective thickness of PQ/PMMA materials, thereby increasing its storage capacity, in 2002, Lin prepared PQ/PMMA materials with thicknesses up to 2.5 cm for the first time by separating thermopolymerization and photopolymerization [89]. Experiments show that the material prepared by this preparation method has the characteristics of low shrinkage and large volume.

That same year, Hsu et al. characterized the prepared PQ/PMMA photopolymers [90], mainly studying the optical properties and scattering effects of PQ/PMMA materials, and used dynamic range to evaluate the criteria for holographic recording. The experiment achieved the single-point multiplexing of 355 holographic gratings and measured 7 mm thick PQ/PMMA materials with a dynamic range up to 14. Experiments show that as a thick-volume holographic material, PQ/PMMA has a low shrinkage rate, indicating the application potential of PQ/PMMA in bulk holographic data storage.

In 2003, Lin performed a chemical analysis of the photoreaction process in the PQ/PMMA holographic record [91]. Mass spectrometry and PL spectroscopy showed that under exposure, the C = O bond of PQ can directly react with the C = C bond of MMA to generate photoproducts. Then, Lin proposed that the holographic recording in PQ/PMMA photopolymers is mainly due to structural changes in PQ molecules, which lead to changes in the refractive index of materials.

Hsiao further studied the PQ/PMMA photoreaction mechanism [72] and used Fourier transform infrared spectroscopy (FTIR), nuclear magnetic resonance spectroscopy (NMR), and gel permeation chromatography (GPC) to identify the molecular structure of photoproducts in PQ/PMMA. The results showed that under exposure, PQ can react with MMA to generate the photoproduct PQ-MMA.

In 2005, Veniaminov studied the PQ/PMMA holographic grating evolutionary characteristics after exposure [92] and proposed that the time evolution process of the grating can be divided into four stages:

(1) The photosensitizer absorbs the photons to be excited to form free radicals and reacts with macromolecules to produce photoproducts, which results in nonlinear changes in gratings,
(2) The spread of PQ molecules from dark to bright areas during exposure enhances holograms,
(3) The movement of the polymer chain is limited by space, causing rapid partial attenuation of the grating,
(4) The diffusion of large molecules in the later stage leads to gentle decay of the grating. This analysis provides an important basis for in-depth study of the photochemical reaction process of polymer samples.

In 2006, Sun studied the effect of temperature on PQ/PMMA materials in holographic recording [93]. Experiments showed that the maximum diffraction efficiency of high-temperature polymerization samples decreases with increasing temperature. For low-temperature polymerization samples, the maximum diffraction efficiency increases and then decreases with temperature within a certain range. Sun used the diffusion equation for the first time to explain the interaction dynamics in the heat treatment process.

Following this work, in 2010, Yu further analyzed the holographic storage stability of PQ/PMMA photopolymers [94]. According to Yu, the stability of the material includes two continuous processes, dark enhancement and hologram attenuation, corresponding to the diffusion of PQ and its photoproducts, respectively. Yu proposed that temperature is the most important parameter for long-term stability, so low temperature and uniform exposure methods are effective ways to improve storage stability.

In 2013, Qi et al. proposed a non-local photopolymerization-driven diffusion (NPDD) model of PQ/PMMA [95] that gives the temporal and spatial variations in the concentrations of chemical components in PQ/PMMA. The model includes:

(1) the time-dependent photon absorption of PQ molecules, including single excited 1PQ* molecules,
(2) recovery/regeneration and bleaching of excited PQ molecules,
(3) non-local effects, and
(4) the diffusion effects of ground- and excited-state PQ molecules and methyl methacrylate (MMA). Subsequently, they applied the model to fit experimental data from PQ/PMMA layers containing three different initial PQ concentrations to test the validity of the proposed model and verify the theoretical results [96].

Not only do PQ/PMMA photopolymers have the recording characteristics of traditional intensity holography, their polarization characteristics too have been studied by many scholars. In 2009, Trofimova et al. first discovered the photoinduced birefringence phenomenon in PQ/PMMA materials [62]. Infrared spectroscopy showed that this phenomenon was caused by a photochemical reaction between the PQ molecule and the substrate, and they also found that the size of the birefringence was mainly related to the doping concentration and temperature of the system. This study laid a foundation for the study of PQ/PMMA materials in polarization holography.

In 2010, Chuang et al. studied the photoinduced birefringence values of PQ/PMMA materials in response to different polarized lights [97], and experimental results showed that PQ/PMMA materials are more sensitive to linear polarized light than circular polarized light, and their photoinduced birefringence can reach 10-5.

In 2011, Lin studied the photoinduced birefringence of 2 mm thick PQ/PMMA photopolymers [98]. When the material is exposed to a linearly polarized beam of 514 nm, its photoinduced birefringence can reach a value of 1.2 × 10^−5^, successfully recording the volume polarization hologram. However, the experimental results also showed that when using circularly polarized light, the maximum diffraction efficiency of the hologram can reach 40%.

PQ/PMMA photopolymers show great potential in holographic recording due to their unique advantages, such as long-term stability [46] (≈100 years), negligible volumetric shrinkage, low price, and polarization properties, but their diffraction efficiency, photosensitivity, and refractive index modulation system are still insufficient. Table 1 presents some characteristic parameters of several common materials used for holographic recording. It is worth noting that the definition of photosensitivity is not the same for different systems, and it is not meaningful to simply compare the number of photosensitivity. Researchers are now committed to further improving the holographic properties of materials through various means. This study mainly introduces the following ways to improve material properties:

### 8.1. Doped Comonomers

Among the many modification methods, the incorporation of copolymer monomers into the substrate MMA of PQ/PMMA photopolymer is a means to effectively improve the material’s holographic properties. Since only a little amount of an MMA monomer can react with PQ to produce photoproducts in a photoreaction, the introduction of monomers with similar structures to MMA can increase the number of vinyl functional groups on the monomer, further increasing the ability of monomer molecules to combine with PQ radicals to produce photoproducts, thereby improving the material’s diffraction efficiency and sensitivity.

In 2006, Lin doped PQ/PMMA with two different monomers: trimethylolpropane triacrylate (TMPTA) and 2-phenoxyethyl acrylate (PEA) [101]. The TMPTA molecule has three vinyl groups, while the PEA molecule has an additional benzene-side functional group. Both have a vinyl structure similar to that of MMA. Experiments showed that the dynamic range of the materials doped with PEA and TMPTA increased by 1.5 times (from ≈0.62 to 1.06) and 2 times (from ≈0.62 to 1.2), respectively, and the sensitivity after doping also increased by 3 times (0.03 to 0.1 cm^2^/J) and 2 times (0.03 to 0.074 cm^2^/J), respectively. For the first time, experiments demonstrated the feasibility of improving holographic properties by doping with monomers with a vinyl structure similar to that of MMA. 

The following year, Krul introduced acrylic acid (AA) into methyl methacrylate (MMA) by copolymerization and prepared modified PMMA film [102]. Experiments showed that using this improved PMMA produces a high thermally stable grating and improves the diffraction efficiency.

In 2011, Yu et al. further doped PQ/PMMA materials with acrylic acid (AA) and methyl methacrylate (MMA) to produce copolymer substrates, improving the thermal stability of the materials [103]. Experiments showed that when the material is doped with 5 mol% AA molecules, the photoproduct diffusion coefficient can be reduced from 0.6 × 10^−18^ to 1.9 × 10^−21^ m^2^/s. At the same time, the increase in refractive index coefficient shows that the AA molecule and the MMA molecule directly produce copolymerization and form a high-refractive-index and high-molecular-weight photoproduct, so the photoproduct is more stable than the sample without the AA copolymer.

In the same year, Ko incorporated poly-square acid-base (3-octylpyrrole-co-square acid) (PSQ3) into PQ/PMMA to improve its holographic data storage characteristics [104]. Polysquaraines possess consecutive donor/acceptor repeating units in the polymer chain, which have considerable charge-transfer interaction and can be used to influence the photoreaction between PQ and MMA, enhancing the holographic properties. Experimental results showed that the doped 0.1% PSQ3 improves the material’s diffraction efficiency and dynamic range (*M*^#^), where the maximum diffraction efficiency is increased from 9.0% to 54.8%, and the value of *M*^#^ is increased from 0.46 to 1.05.

The solubility of PQ is lower in the PQ/PMMA photopolymer, which greatly affects the material’s holographic properties. In 2018, Fan et al. doped PQ/PMMA with tetrahydrofurylate methacrylate (THFMA) to improve the PQ solubility [105], which greatly improved the polarized holographic performance of PQ/PMMA. Specifically, when the ratio of MMA:THFMA is 7:3, the solubility of PQ can be increased from 0.7 to 1.3 wt %, the polarization diffraction intensity is doubled, and the photoinduced birefringence is increased from 10^−5^ to 10^−4^.

Subsequently, they doped PQ/PMMA with N-acryloylmorpholine (ACMO) monomers to improve the diffraction efficiency and photosensitivity sensitivity of the material [106]. ACMO has both a benzene ring and a vinyl structure, so it can improve the solubility of PQ. PQ solubility increased from 0.7 to 1.8%, and experiments proved that the concentration of the photosensitizer PQ greatly affects the material’s holographic properties. When doped with 20% ACMO monomer, the diffraction efficiency and photosensitive sensitivity of PQ/PMMA is increased by six and four times, respectively.

Later that year, Fan et al. also introduced a composite base benzyl methacrylate (BzMA) to optimize the optical properties of PQ/PMMA materials [107]. By mixing BzMA and MMA in a weight ratio of 3:7, they prepared a high concentration of PQ-doped P (B3M7). At 60 °C, the solubility of PQ can be increased from 1.0 to 2.0 wt %. When the PQ concentration in PQ/B3M7 is 1.15 wt %, PQ/P (B3M7) has the highest diffraction intensity and the fastest response speed, and the refractive index modulation increases from 6.3 × 10^−5^ to 9.5 × 10^−5^.

**Figure 16:**
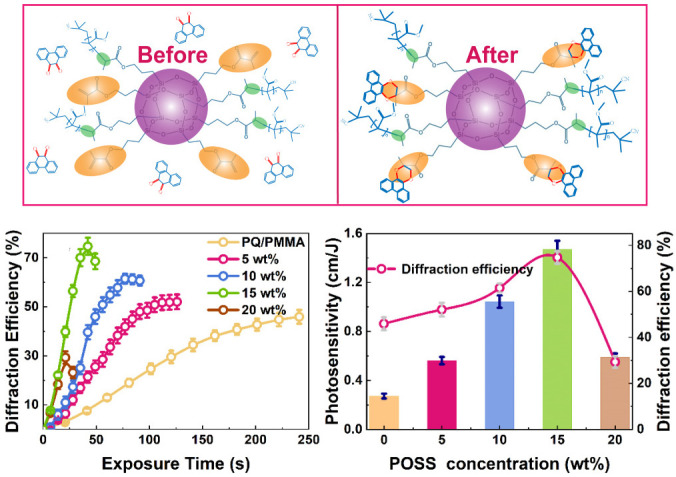
POSS-doped PQ/PMMA reaction mechanism and holographic properties [108].

To further increase the C = C double bond content in the PMMA matrix, in 2022, Hu prepared a star-type polymer POSS–PMMA/PQ by introducing octamethylacryloyloxy polysesquisiloxane (Ma-POSS) into PQ/PMMA photopolymers [108]. Ma-POSS has eight methacryl (Ma) branches and can greatly increase the C = C double bond concentration in the matrix. As shown in Figure 16, an increase in the POSS doping concentration improves the diffraction efficiency and photosensitivity of the material. In particular, when the doping POSS concentration is 15%, the diffraction efficiency and photosensitivity of the material reach the highest value (75% and 1.5 cm/J, respectively). Experiments showed that overbranched star-type polymer POSS–PMMA can significantly improve PQ/PMMA sensitivity (≈5.5 times) and diffraction efficiency (≈50%) and suppress the volume shrinkage by over four times. Analysis showed that not only does POSS participate in thermal polymerization to form star-shaped polymers but also a large number of residual vinyl groups on its branched chain are more likely to react with PQ, which can significantly improve the photoreaction efficiency of POSS–PMMA/PQ.

Relying on the measures of doping comonomers to improve the holographic properties of PQ/PMMA is somewhat effective. On the one hand, the introduction of comonomers can increase the vinyl content of the substrate and provide more C = C double bonds to accelerate the production of photoproducts. On the other hand, doping monomer components that are miscible with PQ can increase the solubility of PQ to a certain extent, and the increase in photosensitizers will also accelerate the photoreaction, improving the sensitivity and diffraction efficiency of materials. In addition, the charge transfer during the photoreaction between the excited-state PQ and the monomer in the photoreaction stage may be an important link for improving the photoinitiation system in the future.

### 8.2. Nanoparticle Doping

In addition to doped comonomers, nanoparticles have been widely studied by scholars due to their unique properties. Studies have found that by adjusting the proportion of incorporation, the refractive index modulation system of PQ/PMMA materials can be improved, and incorporating nanoparticles can improve the stability of the material and reduce the shrinkage rate.

In 2002, Suzuki et al. first doped methacrylate photopolymer films with TiO_2_ nanoparticles and prepared nanoparticle-doped photopolymer materials of 50 μm [109]. Then, they tested the holographic properties of the materials and found that with an increase in nanoparticle concentrations, diffraction efficiency and recording sensitivity increase significantly. Volumetric shrinkage was also suppressed during holographic exposure. As a result, researchers are increasingly focusing on improving the holographic properties of materials by doping with nanoparticles.

In 2005, Tomita et al. studied the mass transfer of nanoparticle-dispersed photopolymers during holographic recording [110]. By directly observing the microscopic structure of the recorded hologram and optically measuring the phase shift between the interference pattern and the recorded hologram, the experiment found that the nanoparticles can be completely transferred from the bright area to the dark area during the exposure process, thus forming a high-contrast hologram [111].

In the same year, Tomita et al. also studied the diffraction characteristics of hyperbranched polymers (HBPs) as transporting organic nanoparticles dispersed in photopolymers and experimentally prepared a transmitted hologram on 51 μm sample films with a diffraction efficiency of nearly 100% [112]. Subsequently, they found that doping with inorganic nanoparticles [113] or organic nanoparticles (hyperbranched polymers) can improve the holographic properties of photopolymers, but the size and aggregation of inorganic nanoparticles will be accompanied by different degrees of holographic scattering, and the use of smaller-diameter nanoparticles will greatly reduce the holographic scattering.

In addition to doping with nanoparticles, in 2005, Lin successfully improved the holographic properties of photopolymers by adding an appropriate concentration of ZnMA to the PQ/PMMA photopolymer [114,115]. Experimental results showed that a small number of ZnMA molecules can be used as catalysts to accelerate the light reaction of PQ molecules. Specifically, the time for the material with a thickness of 600 um to reach the maximum diffraction efficiency was reduced from 102 to 59 s, and the diffraction efficiency was increased from 0.57 to 1.62%.

In 2009, Yu-Fang Chen improved the holographic properties of PQ/PMMA polymers by introducing five different lanthanide metal–organic compounds [116] (Ce^3^^+^, Nd^3+^, Er^3+^, Yb^3+^, and Lu^3+^). The experimental results showed that the doping of PQ/PMMA photopolymer systems by lanthanide metal–organic compounds could improve the material’s diffraction efficiency and dynamic range. The amount of improvement in holographic properties by the compounds was in the following order: Lu^3+^ > Yb^3+^ > Er^3+^ > Nd^3+^ > Ce^3+^. 

In 2011, Hata prepared nanocomposite photopolymer materials based on mercapto-ene systems [117]. Experiments showed that mercapto-oleene photopolymerization can reduce the polymerization effect of oxygen, slowing the growth of molecular chains during the polymerization process, thus delaying the production of gel phenomena. Therefore, the conversion rate of the monomer of the system is relatively high, and the shrinkage of the material can be reduced.

In the same year, Yu experimentally verified the effect of doping PQ/PMMA materials with SiO_2_ nanoparticles on the holographic recording grating [118], indicating that doping with SiO_2_ nanoparticles can make the order of photochemical reactions nonlinear and improve the formation speed of the grating. The diffusion of PQ molecules and the de-diffusion of SiO_2_ nanoparticles are the main photochemical reaction processes after exposure.

In 2014, Li doped PQ/PMMA materials with gold nanoparticles to improve the photoinduced birefringence of the material [65]. Compared with PQ/PMMA materials, the linearly polarized photobirefringence of AuNP–PQ/PMMA materials with 1 mm thickness increased from 4.2 × 10^−5^ to 5.8 × 10^−5^, which was an increase of about 38%.

Peng Liu et al. doped PQ/PMMA with nanolevel silver and prepared a new photopolymer material [119]. Through ultra-fast nanosecond laser holographic exposure, they elaborated the grating formation of silver-nanopolymer-doped photopolymer nanocomposites and improved photopolymer holographic properties. The optimized polymer diffraction efficiency was as high as 51.4%.

In 2019, Ying Liu used liquid-phase laser ablation technology to prepare a PQ/PMMA material doped with Al nanoparticles [81] and investigated the properties of intensity holography and polarization holography. Compared with the PQ/PMMA material without doping nanoparticles, the diffraction efficiency of the intensity holography was increased by 57.15% and the polarization diffraction efficiency was also improved (from 0.6% to 4.6%).

Ying Liu used orthogonal polarization to study the polarization properties of PQ/PMMA doped with SiO_2_ nanoparticle (NP) [120]. On introducing SiO_2_ nanoparticles into a colloidal solution of methyl isobutyl ketone (MIBK) as a comonomer, the concentration of PQ in the system increased to 1.2 wt %. Experiments showed that with the dispersion of the SiO_2_ nanoparticles in the colloidal solution and the increase in PQ concentration, the diffraction efficiency and photoinduced birefringence are exponentially increased.

Yu Xin Chen greatly improved the polarization properties of the material by doping PQ/PMMA photopolymers with graphene (GO) [121]. Experiments showed that GO will not only induce polymerization and grafting on the surface of the polymer but also adsorb photosensitizers to aggregate around the polymer, thereby promoting photopolymerization. In addition, the deep correlation between the material polarization holographic properties and the material molecular weight was revealed for the first time.

**Figure 17:**
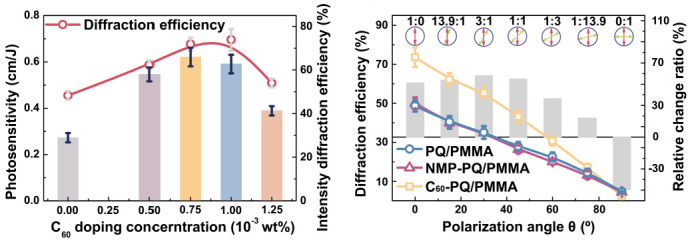
Properties of C_60_-doped PQ/PMMA holography [46].

In 2022, Hu significantly improved the diffraction efficiency and photosensitive sensitivity of intensity holography but reduced the polarization sensitivity of PQ/PMMA photopolymers by introducing fullerene (C_60_) into PQ/PMMA photopolymers [46]. Experimental analysis showed that due to the strong π–π interaction between C_60_ and PQ, the deflection of PQ molecules under polarized light exposure will be limited, reducing the photoinduced birefringence of C_60_–PQ/PMMA materials. The results are shown in Figure 17.

Nanoparticle doping relies on the inter-diffusion model of monomers and nanoparticles to improve the refractive index modulation of PQ/PMMA and has been widely studied by scholars. Past studies have shown that this improvement method is effective. However, the uniformity of dispersion of nanoparticles in materials cannot be ignored, although there are improved measures to add organic solvents to improve their uniformity. In addition, due to the low solubility of nanoparticles, they still have some limitations in improving the holographic characteristics of materials.

### 8.3. Replace the Photosensitizer

Since the solubility of the photosensitive agent phenanthrenequinone (PQ) in methyl methacrylate (MMA) solution is limited at room temperature, researchers tried to further improve the holographic properties of the material by replacing the photosensitizer.

**Figure 18:**
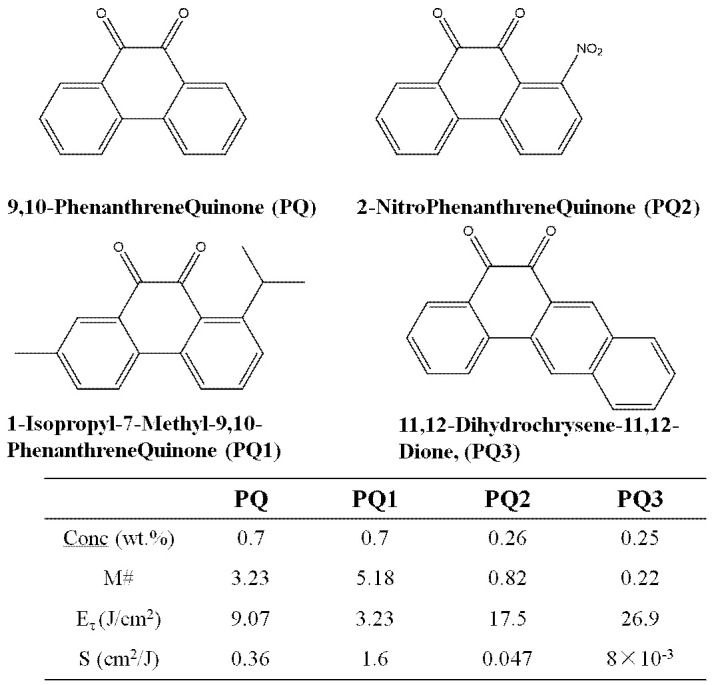
PQ derivatives’ chemical structures and experimental results [122].

In 2008, Lin et al. reported on the influence of different PQ derivative components on the holographic properties of materials [122], introducing phenanthraquinone PQ derivatives containing different functional groups in the side chain, as shown in Figure 18. A series of PQ/PMMA, PQ1/PMMA, PQ2/PMMA, and PQ3/PMMA photopolymerizations were prepared, and the experimental results showed that the holographic performance of PQ1/PMMA was better. It was also found that the functional group of the electron supply can effectively improve the holographic recording performance.

In 2009, Hsiao et al. used photosensitizer Irgacure 784 to replace PQ and conducted comparative experiments [123]. Experimental results showed that compared with the PQ photosensitizer, the Irgacure 784 photosensitizer has a strong absorption at 532 nm and can improve the photosensitivity of the material.

In 2013, Ando et al. synthesized a polymer PSO_3_ and co-doped PMMA with PQ and the polymer PSO_3_ [124]. The experiment showed that a small amount of PSO_3_ can greatly improve holographic performance, with the maximum diffraction efficiency reaching 54%. They also synthesized a new photodegradable aromatic ketone derivative AK1, which is more than 20 times more soluble in MMA than the PQ molecule, and AK1 is a photosensitive material whose photoinduced birefringence is more than four times that of the PQ/PMMA material.

In 2017, Ying Liu further replaced the photosensitizer PQ with Irgacure 784 (TI) and set up a control experiment [125]. Experiments showed that under the same conditions, the saturated refractive index modulation and sensitivity of the sample Irgacure 784/PMMA are better than those of the sample of PQ/PMMA, and the diffraction efficiency of Irgacure 784/PMMA is more than three times that of PQ/PMMA under the same exposure time. 

Later, Peng Liu used an optimized three-step thermal polymerization method to prepare a novel cationic photoinitiator: dimethene dispersed polymethyl methacrylate (Ti/PMMA) photopolymer [126,127]. The experiments studied detail the effect of changes in material thickness on holographic properties. An experiment achieved a response time of 4.98 s on 1 mm TI/PMMA, while a cumulative grating intensity of 6.88 and a single grating diffraction efficiency of 74% was achieved on 3 mm TI/PMMA. Subsequently, the polarization holographic properties of TI/PMMA polymers under orthogonal linear polarization exposure were also verified [128], with diffraction efficiencies of 1, 2, and 3 mm TI/PMMA polymers being 5%, 12%, and 16%, respectively.

Changing the photosensitizer is another attempt to improve the holographic characteristics of materials. Although researchers have carried out relatively little work in this area, perhaps finding a photosensitizer with a higher solubility and a faster photoreaction rate than those of PQ in PMMA matrix can solve the current problem of insufficient diffraction and photosensitivity of the materials.

### 8.4. Optimize Preparation Conditions

In addition to doping with copolymers, doping with nanoparticles, and altering photoinitiators to improve the material’s holographic properties, researchers are working to optimize the preparation conditions of PQ/PMMA to further optimize its performance.

In 2010, Hong Peng Liu increased the prepolymerization reaction temperature to 60 °C [129], thereby increasing the mass fraction of PQ in the system, and the solubility of PQ increased from 0.7 to 1 wt %.

Song discussed three reasons for the occurrence of bubbles when preparing photopolymer PQ/PMMA materials through analysis and simulation:

(1) The thermal initiator AIBN decomposes to produce nitrogen, and during the polymerization process, the liquid sample gradually becomes viscous, resulting in the inability of the bubbles to be discharged,
(2) When MMA is converted into PMMA, the energy released by the formation of σ bonds is greater than the energy absorbed when the π bonds break and the boiling point of MMA is low, so MMA boiling produces bubbles,
(3) When MMA is completely converted into PMMA, there are different degrees of shrinkage, and when the sample solidifies, air will be sucked in at the edge of the mold due to negative pressure and bubbles will be generated. The experimental analysis can act as a guide in PQ/PMMA material preparation.

**Figure 19:**
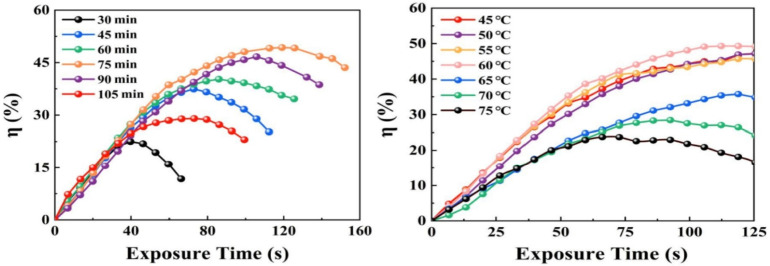
Exposure-time-dependent diffraction efficiency of PQ/PMMA with different stirring times and at different temperatures [99].

In 2021, using the grid method, Xi Chen analyzed the holographic properties of PQ/PMMA prepared in various conditions in terms of time and temperature during the mixing and baking polymerization stages of the pre-polymerization process [99], determining the optimal preparation conditions for photopolymers. The experimental results are shown in Figure 19. When the pre-polymerization stirring temperature is set at 60 °C and the stirring time is 75 min, material with the best diffraction efficiency can be obtained

In this study, we mainly described the research origin and development processes of PQ/PMMA photopolymers. PQ/PMMA materials have some inherent characteristics that make them suitable for holographic recording, such as a low price, a large thickness, and a simple preparation process. However, their diffraction efficiency and photosensitive sensitivity, both of which are low, still need to be improved. We also introduced several different improvement measures that partly improve the holographic characteristics of PQ/PMMA. 

## 9. Conclusions

Since PQ/PMMA photopolymers were proposed, they have received great attention in holographic recording, especially in holographic data storage. In addition, PQ/PMMA can be used for the generation of optical components, such as polarizers [130], vector light fields [68], and vortex beams [131]. In this paper, we reviewed the development of PQ/PMMA and described its thermal and photoreaction processes and holographic characteristics in detail. To optimize the practical application of PQ/PMMA in holographic recording, it is essential to further improve the holographic performance of PQ/PMMA. Several improvement methods were proposed, including:

(1) doping by comonomers,
(2) introduction of nanoparticles,
(3) replacement of the photosensitizer,
(4) and optimization of preparation conditions.

These measures led to a focus on improving the sensitivity and refractive index modulation of PQ/PMMA. According to previous studies, it is worth paying attention to finding new photoinitiation systems and introducing high-refractive-index comonomers to improve the holographic characteristics of PQ/PMMA in the future.

## Figures and Tables

**Table 1:** Characteristic parameters of several holographic recording materials.

Holographic Recording Material	Diffraction Efficiency	Photosensitivity	Thickness	Recording Wavelength (Power)
0.1PQ-PQ/PMMA [99]	≈50%	≈0.3 cm/J	≈1.5 mm	532 nm (100 mw/cm^2^)
PVA/AA [36]	≈94%	≈8 mJ/cm^2^	≈60 um	632 nm (1 mw/cm^2^)
Epoxy–Resin [40]	≈92%	≈11.7 × 10^−3^ cm^2^/J	≈0.25 mm	532 nm (2 mw/cm^2^)
HPDLCs [100]	≈82%	2.3 cm/mJ	Liquid	442 nm (8.8 mw/cm^2^)

## Data Availability

Not applicable.

## Sample Availability

Samples of the compounds are not available from the authors.
